# Microbiota–Gut–Brain Axis: Mass-Spectrometry-Based Metabolomics in the Study of Microbiome Mediators—Stress Relationship

**DOI:** 10.3390/biom15020243

**Published:** 2025-02-07

**Authors:** Nicolò Interino, Rosalba Vitagliano, Federica D’Amico, Raffaele Lodi, Emanuele Porru, Silvia Turroni, Jessica Fiori

**Affiliations:** 1 IRCCS Institute of Neurological Sciences of Bologna, 40139 Bologna, Italy; nicolo.interino@ausl.bologna.it (N.I.); rosalba.vitagliano@ausl.bologna.it (R.V.); raffaele.lodi@ausl.bologna.it (R.L.); 2 Department of Pharmacy and Biotechnology, University of Bologna, 40126 Bologna, Italy; federica.damico8@unibo.it; 3 Occupational Medicine Unit, Department of Medical and Surgical Science, Alma Mater Studiorum, University of Bologna, 40138 Bologna, Italy; emanuele.porru2@unibo.it; 4 Department of Chemistry “G. Ciamician”, University of Bologna, 40126 Bologna, Italy

**Keywords:** microbiota–gut–brain axis, mass spectrometry, stress, mediators, metabolomics

## Abstract

The microbiota–gut–brain axis is a complex bidirectional communication system that involves multiple interactions between intestinal functions and the emotional and cognitive centers of the brain. These interactions are mediated by molecules (metabolites) produced in both areas, which are considered mediators. To shed light on this complex mechanism, which is still largely unknown, a reliable characterization of the mediators is essential. Here, we review the most studied metabolites in the microbiota–gut–brain axis, the metabolic pathways in which they are involved, and their functions. This review focuses mainly on the use of mass spectrometry for their determination, reporting on the latest analytical methods, their limitations, and future perspectives. The analytical strategy for the qualitative–quantitative characterization of mediators must be reliable in order to elucidate the molecular mechanisms underlying the influence of the above-mentioned axis on stress resilience or vulnerability.

## 1. Introduction

The bidirectional communication system between the gastrointestinal tract and the brain was originally termed the “gut–brain axis” and has recently been renamed the “microbiota–gut–brain axis” [1], highlighting the critical role of the gut microbiota (GM) in maintaining host homeostasis and suggesting that these microorganisms actively participate in shaping and maintaining host physiology almost as an additional organ [2]. The microbiota–gut–brain (MGB) axis can influence host functions in a variety of ways, involving immune, neural, endocrine, and metabolic pathways. The enteric nervous system (ENS), as well as the sympathetic and parasympathetic autonomic nervous systems, play an important role in communication within this axis. Indeed, perturbations of the gut microbial ecosystem, also known as dysbiosis, which can be due to various endogenous and exogenous stressors, may lead to impairment of the intestinal barrier, alterations in its motility, and potential activation of immune responses. Nowadays, a number of pathways and potential mechanisms that may regulate microbiota–brain interactions are under investigation [3]. Gut microbes can alter the central neurotransmitter balance by producing neurotransmitters themselves or their precursors [2]. These chemical signals can act locally by interfering with host synthesis and, by crossing the gut mucosal layer, influence peripheral systems and potentially the central nervous system (CNS). In this context, GM alterations have been implicated in the development of stress, anxiety [1,2,4,5], depression [6,7], autism [8,9], neurodegeneration [10,11,12], and stroke/brain injury [13].

With regard to stress, it is well known that it negatively impacts the health of the intestine and, of course, through the exchange of active molecules via the MGB axis, the CNS. Genetic and environmental factors influence individual responses to stress and, more recently, a role for GM in regulating stress-related changes has been demonstrated [2]. However, the mechanisms underlying this reciprocal influence remain largely elusive and numerous studies are currently aimed at discovering or validating robust biomarkers of susceptibility to stress.

Understanding the neurobiological mechanisms underlying the influence of GM on brain function and behavior has become a key research priority. In particular, it is essential to unravel the mediators involved in the cross-talk along the MGB axis. In this context, mass spectrometry (MS) combined with liquid chromatography (LC) and gas chromatography (GC) has emerged over the last decades as the technique of choice for the identification and quantification of metabolites involved in numerous metabolic pathways and in different biological matrices [14,15,16]. Thanks to its ability to scan ionic molecules based on their mass/charge ratio in a magnetic field and to fragment structures, MS is a very powerful and versatile analytical technique for the characterization of metabolic profiles over a wide range of chemical diversity and concentrations. In the framework of the so-called omics sciences, the analysis of metabolites, metabolomics, is of considerable importance as it is the bottom of the omics cascade and represents the endpoint of changes in gene expression, transcription, and protein production from the host counterpart, as well as metagenomics and metatranscriptomics for the full characterization of GM in the MGB axis. As mentioned before, MS combined with separation techniques is currently the most powerful analytical technique in the study of metabolic pathways at the molecular level [14,15,16]. Thanks to its characteristics, it contributes to filling the gaps in knowledge of the biological mechanisms underlying neurological diseases. Untargeted MS is able to verify the presence and identity of already known molecules and also plays a fundamental role in the biomarker discovery of new mediators. Thanks to the identification of new metabolites and the differential quantitative analysis of mediator expression by untargeted and targeted MS, a great contribution can be made in elucidating the microbiota–gut–brain axis communication. Furthermore, MS, by identifying molecules involved in pathogenetic mechanisms, is able to recognize and monitor diagnostic and disease progression biomarkers and, also, the targets of therapeutic interventions.

In this review, we briefly introduced the various mechanisms underlying the bidirectional microbiota–gut–brain axis in relation to stress, anxiety, and depression, highlighting its complexity. We then described the primary mediators and the metabolic pathways involved, categorizing them according to their chemical class and/or biological function. Following this introduction, the review focused on the main topic: the analysis of metabolites using mass spectrometry. In particular, we discussed both targeted and untargeted approaches, emphasizing their unique characteristics, advantages, disadvantages, and the sample preparation methods for different biological samples, such as feces, plasma, urine, and others. We aimed to highlight the studies conducted so far to identify small molecules, as metabolites, involved in the MGB axis under stress conditions, using separation techniques combined with MS. The review covered steps from the treatment of different biological samples to MS analysis, including multi-omics data integration. In an effort to ensure a comprehensive search, as suggested by the literature, multiple databases were examined [17].

## 2. Microbiota–Gut–Brain Axis in Stress

### 2.1. The Bidirectional Microbiota–Gut–Brain Axis

The MGB axis is a complex bidirectional communication system involving multiple interactions between the gut and the brain. Several communication pathways have been identified, including neuroimmune pathways, activation of afferent sensory fibers of the vagus nerve, and neuroendocrine pathways [18,19,20,21]. In particular, the GM has been associated with various effects, such as (i) the synthesis of neuroactive microbial products, such as short-chain fatty acids (SCFAs) derived from the fermentation of complex polysaccharides by the GM, which play a key and multifactorial role in human physiology [22], as well as norepinephrine (NE), dopamine (DA), gamma-aminobutyric acid (GABA), serotonin (5-HT), nitric oxide, and other microbial-derived molecules; (ii) the stimulation of hormone release (e.g., 5-HT) by enteroendocrine cells; (iii) the induction of cytokine release by mucosal immune cells; and (iv) the direct activation of nerve fibers, including the vagus nerve [23,24]. With respect to the latter, Toll-like receptors (TLRs) that recognize microbe-associated molecular patterns (MAMPs), such as lipopolysaccharide, peptidoglycan, flagellin, lipoteichoic acid, and/or DNA/RNA molecules, have been identified on dorsal root ganglia, suggesting that the GM and/or its components may directly interact with nerve fibers [25]. Gut microorganisms may also regulate host production of metabolites and neurotransmitters involved in gut–brain signaling, including (i) oxytocin, which enhances social behavior and whose plasma and brain levels have been shown to increase in mice following treatment with *Lactobacilli*, particularly *Lactobacillus reuteri*; (ii) brain-derived neurotrophic factor (BDNF), which promotes neuronal plasticity in the ENS and alleviates anxiety and depression-like behaviors in mice, with levels upregulated by *Bifidobacterium* spp.; and (iii) 4-ethylphenylsulfate (4-EPS), the reduction in which by *Bacteroides fragilis* has been associated with improvements in anxiety-like behavior, repetitive behavior, and communication deficits in mice [26].

Through the production of SCFAs, release of MAMPs, increased cytokine levels, and other mechanisms, GM can also modulate the hypothalamic–pituitary–adrenal (HPA) axis, the primary neuroendocrine system directly influencing stress adaptation and resilience [27]. In particular, a landmark study in 2004 demonstrated for the first time that germ-free mice exhibit a hyper-reactive HPA axis, leading to increased levels of adrenocorticotropic hormone and corticosterone following exposure to a stressful stimulus [28]. A few years later, Heijtz et al. [29] showed that germ-free mice display a distinct gene expression pattern compared to control mice, particularly in the hippocampal and cortical regions, which likely contribute to the altered activity of the HPA axis. Since then, several studies have confirmed that gut microbes can differentially affect the stress circuitry, establishing the foundation for GM-based precision strategies in the prevention and treatment of stress-related disorders [30,31].

All of the above observations are closely related to the inverse component, namely, the MGB axis [32]. Indeed, stress and emotions can lead to alterations in hormone and neurotransmitter levels, which subsequently influence gut physiology. These changes can directly affect bacterial gene expression and signaling pathways, ultimately altering the composition and function of the GM, including increased intestinal permeability, with cascading effects on host health. For example, catecholamines can induce the growth of microorganisms with pathogenic potential, such as *Escherichia coli*, *Yersinia enterocolitica*, *Salmonella enterica*, and *Pseudomonas aeruginosa*, and promote biofilm formation by *Staphylococcus* [33].

A body of research, primarily conducted in animal models, has demonstrated the involvement of the GM in the development of the CNS, particularly in the following processes: formation and integrity of the blood–brain barrier [34]; neurogenesis [35,36]; maturation and branching of microglia [37]; myelination [38,39]; development and functionality of the HPA axis; and neuronal survival, growth, and differentiation through the expression of neurotrophins [40,41,42], neurotransmitters, and their receptors [42,43].

It is therefore not surprising that GM alterations (e.g., dysbiosis) have been linked to the development of neurological disorders, including autism spectrum disorders, anxiety, depressive-like behaviors, epilepsy, neurodegenerative disorders, and others, the onset of which is often preceded or accompanied by gastrointestinal symptoms [23]. In particular, several studies agree in identifying the following common features of dysbiosis: (i) reduced diversity, with only a few species present and poorly interconnected; (ii) loss of microorganisms typically associated with health and generally SCFA producers, such as those from the *Lachnospiraceae* and *Ruminococcaceae* families; and (iii) enrichment of pathogens or pathobionts, including mucus-degrading bacteria, such as *Ruminococcus torques*, *Ruminococcus gnavus*, and *Akkermansia muciniphila*, with a potential loss of intestinal barrier integrity [44].

With specific regard to stress, the involvement of GM is supported by the wealth of evidence highlighting the benefits of its modulation. For example, a combination of prebiotics, such as fructo-oligosaccharides (FOS) and galacto-oligosaccharides (GOS), has been shown to produce antidepressant and anxiolytic effects in mice exposed to chronic stress [45]. Administration of GOS to healthy volunteers for three weeks suppressed the stress response and enhanced the processing of positive versus negative vigilance [46]. A prolonged diet enriched in ω-3 polyunsaturated fatty acids (PUFAs) and vitamin A showed beneficial effects on cognitive and memory deficits induced by social instability stress, including the normalization of hippocampal BDNF expression levels and GM composition [47]. Traditional probiotics, such as species of *Bifidobacteria* and *Lactobacilli*, have been associated with a range of beneficial effects. These include modulation of resting neural activity in healthy volunteers during social stress (which correlated with increased vitality and reduced mental fatigue), as well as reduction in stress and anxiety in stressed adults through enhancement of the 5-HT pathway [48,49]. In particular, there is considerable evidence for *Lacticaseibacillus paracasei Shirota*, the administration of which has been associated with: (i) a reduction in anxiety symptoms in patients with chronic fatigue syndrome [50]; (ii) attenuation of cortisol hypersecretion and physical symptoms, likely mediated through vagal signaling and reduced stress reactivity, along with the maintenance of sleep quality in students under academic exam stress [51,52]; and (iii) decreased anxiety and stress, as well as improved aerobic capacity, in university badminton players [53]. More recently, next-generation probiotic candidates, such as *Bacteroides uniformis*, *Roseburia inulinivorans*, *Eubacterium rectale*, and *Faecalibacterium prausnitzii*, have been shown to improve mood primarily through three pathways associated with nervous system functions: the production of SCFAs; the regulation of aspartate, glutamate, and tryptophan metabolism (e.g., conversion of glutamate to GABA and of tryptophan to 5-HT, kynurenic acid or tryptamine); and the modulation of taurine and cortisol metabolism [54].

### 2.2. Main Mediators and Pathways Related to Stress

**Figure 1:**
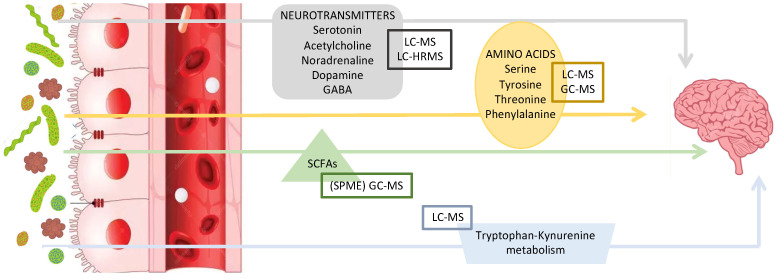
Microbiota–gut–brain axis: main mediators and mass-spectrometry-based analytical techniques for their determination. GABA, gamma-aminobutyric acid; GC, gas chromatography; HRMS, high-resolution mass spectrometry; LC, liquid chromatography; MS, mass spectrometry; SCFAs, short-chain fatty acids; SPME, solid phase microextraction.

Recent MS-based metabolomics has led to significant progress in identifying and quantifying metabolic interactions between the GM and the brain [14]. Below, we discuss the key mediators and pathways of the MGB axis related to stress that are known to date (see also Figure 1). In order to simplify the reading, given the diversity of the metabolites, we have grouped them by chemical class and/or biological activity.

#### 2.2.1. Neurotransmitters

Neurotransmitters are essential molecules for communication within the CNS and the ENS, playing a critical role in the regulation of emotions, behavior, and mental health. In recent years, the interaction between the GM and the nervous system has gathered significant attention due to the capacity of GM to influence the production, metabolism, and function of numerous neurotransmitters. This intricate MGB axis has opened new avenues for understanding the relationship between gut health and neuropsychiatric disorders, such as anxiety and depression [18,21].

Neurotransmitters can be classified into several key categories: monoamines (including both catecholamines, e.g., DA and NE, and indolamines, e.g., 5-HT), gasotransmitters (e.g., nitric oxide, carbon monoxide), purines (adenosine triphosphate and adenosine), amino acids (e.g., glutamate, aspartate, serine, GABA, and glycine), neuropeptides (oxytocin, somatostatin, and opioid), and smaller compounds, such as acetylcholine (ACh).

The monoamine 5-HT is the most studied neurotransmitter in psychiatry and is directly related to GM composition. It contributes to happiness and well-being and is widely present in the platelets, intestines, and brains of mammals [55]. The GM has the ability to promote 5-HT biosynthesis and regulate the permeability of the blood–brain barrier to modulate its distribution and fate in the CNS. Specifically, 5-HT production has been attributed to *Enterococcus*, *Streptococcus*, *Escherichia*, and *Candida* spp. Several studies have shown that depressive-like and anxiety-like changes are associated with decreased 5-HT levels in the hippocampus and prefrontal cortex of animal models exposed to chronic unpredictable mild stress (CUMS) [56,57]. Lv et al. demonstrated that the serotoninergic response, under conditions of acute stress, is influenced by the GM in a sex- and brain-region-dependent manner. However, little is known about how the GM may modulate the host’s serotonergic response following stress exposure [58].

The GM is one of the sources of catecholamines, such as NE produced by *Saccharomyces* and *Bacillus* spp. and DA produced by *Serratia* and *Bacillus* spp. [12,59]. These catecholamines, such as DA and NE, are the major neurotransmitters used by the CNS to mediate several neuroactive functions associated with psychiatric disorders, such as anxiety and depression [20,55,60].

Furthermore, the GM plays a crucial role in the production of amino acids, influencing various physiological and metabolic processes. The metabolic pathways of amino acids, such as serine, tyrosine, threonine, and phenylalanine, play an important role in the development of brain disorders. Changes in the levels of these amino acids in plasma, serum, and urine have been demonstrated in numerous brain disorders, such as autism, schizophrenia, and Huntington’s disease [61,62,63,64,65,66,67,68,69,70,71]. 

The metabolism of tryptophan, the precursor of several neuroactive molecules, including 5-HT, has been extensively studied to establish a potential link for bidirectional communication between the gut and the brain [72]. Most of the tryptophan (90%) is metabolized to kynurenine via the kynurenine pathway while 3% is metabolized to 5-HT and the remainder is converted by the GM to indole and its derivatives, which bind to aryl hydrocarbon receptors and, thereby, modulate the immune response [73,74,75]. The enzyme responsible for tryptophan metabolism is indoleamine-2,3-dioxygenase 1 (IDO1), which converts the neurotransmitter to kynurenine. The latter can be further metabolized to kynurenic acid, a neuroprotective molecule, by the enzyme kynurenine aminotransferase, or to 3-hydroxyquinurenine (3-HK) by kynurenine hydroxylase, which is finally converted to quinolinic acid. In the brain, tryptophan is metabolized to quinoline and quinurenic acid by microglia and astrocytes, respectively. It is known that excessive activation of the IDO1 enzyme can lead to neurotoxicity and increased risk of neuropsychiatric disorders, such as depression [57,75]. Tryptophan can also be transformed by GM directly into indole and its derivatives, such as indole-3-aldehyde (IAld), indole-3-acetic acid (IAA), indole-3-propionic acid (IPA), indole-3-acetaldehyde (IAAld), and indole acrylic acids. In fact, observational studies in animal models of stress and depression have demonstrated that the administration of probiotics, such as *Bifidobacterium* and *Lactobacillus* spp., is able to modulate GM with an effect on colonic 5-HT levels and tryptophan availability [21,57,75].

Another neurotransmitter secreted by GM, particularly the *Lactobacillus* and *Bifidobacterium* species, is GABA. GABA production by the GM occurs mainly during a stress response to protect the body from the acidic environment of the stomach. GABA-producing bacteria have the potential to influence brain function, behavior, and mental health and communicate with the brain via the vagus nerve. However, the exact relationship between GABA and the GM and the brain is not fully understood, for example, it is yet unknown whether GABA crosses the blood–brain barrier. On the other hand, there is evidence suggesting that GM controls GABA-related anxiety symptoms [20].

*Lactobacillus* spp. and *Bifidobacterium* spp. can also produce nitric oxide (NO), which has pleiotropic effects, including on the immune response, vascular control, and neurotransmission [76]. It has been implicated in resistance to stress and in a variety of neuropsychiatric and neurodegenerative diseases [77]. In particular, under pathological conditions, NO can react with superoxide anion to form peroxynitrite, which, in turn, can react with the phenolic ring of tyrosine to form nitrotyrosine, which has been shown to contribute to the onset and progression of neurodegenerative processes, such as Alzheimer’s and Parkinson’s disease.

Moreover, GM influences the production of neuropeptides by enteroendocrine cells (EECs) in the gastrointestinal tract [78]. These neuropeptides, including peptide YY (PYY) and neuropeptide Y (NPY), facilitate communication between the ENS and the CNS, playing a critical role in regulating essential functions, such as digestion, eating behavior, and the stress response [79]. Additionally, oxytocin, a neuropeptide primarily known for its role in regulating emotions, social behavior, and stress responses, has recently emerged as a key player in the gut–brain axis, underscoring the complex interaction between GM and neuropeptides in maintaining physiological and emotional balance [79]. While direct evidence remains limited, several studies suggest that GM may influence oxytocin levels in the brain. For instance, supplementation with probiotics, such as *Lactobacillus reuteri*, has been shown to enhance oxytocin production, leading to improved social behaviors and accelerated wound healing in animal models [80].

Finally, gut microbes have been identified as a crucial component of the ACh production system, which plays essential roles in the ENS and in the CNS of mammals [14]. ACh regulates various functions, including intestinal motility, immune response, sleep, memory, temperature control, and blood pressure. However, its most significant role is in cognitive functions, such as learning and memory [81].

Imbalances in ACh production in the brain are responsible for neurodegenerative diseases, leading to cognitive decline and memory deficits. In this context, interventions targeting the microbiota, such as probiotics or dietary modifications, could support ACh production, thereby improving neurological and cognitive health, especially in conditions characterized by cholinergic dysfunction [18].

#### 2.2.2. Bioactive Lipids

The GM is also involved in the production of several bioactive lipids, such as SCFAs, which are beneficial for brain health and behavior [78]. SCFAs, including acetate, propionate, butyrate, and valerate, are primarily derived from the fermentation of dietary fiber and represent GM metabolites with a strategic role in gastrointestinal homeostasis, as well as metabolic and immune function. In this regard, several studies have reported decreased levels of SCFAs in fecal samples from depressed and/or stressed patients and animal models [18,57]. In particular, butyrate contributes significantly to host health and has been positively implicated in many aspects of brain function and behavior. In contrast, high levels of propionate may have negative effects on mental health and social behavior and may induce brain abnormalities similar to those found in autism [14,18]. The study by van de Wouw et al. demonstrated that treatment with SCFAs ameliorated psychosocial stress-induced changes in reward-seeking behavior and increased reactivity to an acute stressor and intestinal permeability in vivo [19]. In addition, SCFAs showed specific antidepressant and anxiolytic effects in behavioral tests, which were not present when the mice were also exposed to psychosocial stress. The possible mechanism associated with the protective effects of some SCFAs in mental disorders could be the activation of G-protein-coupled receptors (GPCRs). For example, the activation of GCPR43, expressed on enteroendocrine cells, stimulates the release of intestinal peptides, PYY, and glucagon-like peptide 1 from enteroendocrine cells. SCFAs can regulate the expression of tight-junction proteins and regulate brain functions, such as influencing gut–brain hormonal communication [14,18,75].

Furthermore, long-chain fatty acids and PUFAs are involved in many important functions, including neurogenesis, neurotransmission, and neurodevelopment, and have structural and signaling roles. The CNS contains some PUFAs, such as ω-6 and ω-3, and their derivatives, like arachidonic acid and docosahexaenoic acid, which play an important role in the development of a variety of neurodegenerative diseases [14,75].

To date, we know that the GM is able to modulate the production of SCFAs and PUFAs and their metabolites, which are linked to brain function and behavior. SCFAs influence brain neurochemistry by regulating BDNF expression and reducing stress-related hormones like cortisol. Similarly, PUFAs contribute to the synthesis of key neurotransmitters, such as 5-HT and DA, which are essential for mood regulation and social behavior. Alterations in these metabolites can underline conditions like depression and anxiety, highlighting the complex interplay between gut microbial composition and brain signaling pathways [14].

#### 2.2.3. Steroid-Based Molecules

Steroid hormones also play an important role along the MGB axis. It should be emphasized that they are important physiological regulators of the nervous system, in addition to the fact that their levels are differentially influenced by several factors, including age and sex [82]. Therefore, there is a need to better understand how age-related changes in estrogen and androgen levels may influence GM and susceptibility to disease. Furthermore, neuroactive steroids play an important role in inflammatory processes in the brain, which, in turn, modulate behavioral states. In fact, hyperactivity of the main stress axis, the HPA axis, is characterized by high levels of cortisol, one of the steroid hormones derived from cholesterol and secreted by the adrenal gland. Cortisol plays a very important role in the stress response and influences brain size, structure, and function [55,82]. A LC-MS-based metabolomics study showed that cortisol alterations contribute to the development of Parkinson’s disease [14]. Furthermore, it was demonstrated that in post-menopausal women, GM diversity was positively associated with the ratio of estrogen metabolites in urine [82]. Fecal microbiota transplantation from adult male mice to immature female mice was associated with increased testosterone levels and metabolomic changes in the recipient females [82]. Finally, several studies have shown that stress causes long-term alterations in both the diversity and composition of the GM, including an increase in plasma corticosterone. These studies provide some evidence that the GM is influenced by gonadal steroid hormones and that steroid levels may in turn be altered by the GM [14,18,75].

In addition, primary bile acids are known to be modified by GM through deconjugation, dehydrogenation, dehydroxylation, and sulfation reactions to form secondary bile acids, such as ursodeoxycholic acid or tauroursodeoxycholic acid, and oxo-bile acids [83,84] are considered neuroprotective as they reduce neuronal loss and the accumulation of toxic aggregates in different experimental models of neurodegenerative diseases [14].

## 3. Metabolite Determination: From Sample Treatment to MS Analysis

An increasing number of studies have reported the use of metabolomics as a powerful tool for the identification and, when possible, quantification of metabolic interactions between GM and the host brain [14,16,18,56,78,85,86,87]. The study of metabolites by MS is able to monitor not only mediators but also molecules identified as diagnostic biomarkers and/or disease progression and therapeutic targets.

In the field of metabolomics, the most widely used analytical techniques are NMR (Nuclear Magnetic Resonance) and MS, with MS being the predominant choice. Despite the destructive nature of mass spectrometric analysis and its limitations in the structural elucidation of unknown compounds, MS offers unparalleled sensitivity and specificity. This is particularly true when MS is coupled with separative techniques, such as GC and/or LC. Furthermore, MS enables a relatively straightforward automation process. Together, these advantages allow for faster, deeper, and thus more efficient analyses [73,78,88]. 

The main steps in metabolome investigation include the pre-treatment of complex metabolite mixtures by sample lysis, if necessary, and purification of the extracted metabolites from the samples of interest. The metabolite separation is usually performed by high-performance LC (HPLC) or GC, allowing the analysis of a wide range of metabolites using high-sensitivity MS platforms. The final step is metabolite identification and subsequent quantification, which is calculated based on the mass spectral fingerprint and MS fragmentation pattern [78,88].

However, it must be emphasized that the analysis of metabolites involved in the MGB axis is a challenging issue, especially for the choice of biological matrix. It is known that each biological matrix, such as plasma, feces, or cerebrospinal fluid, carries different metabolic information and requires different sample treatments [20].

**Figure 2:**
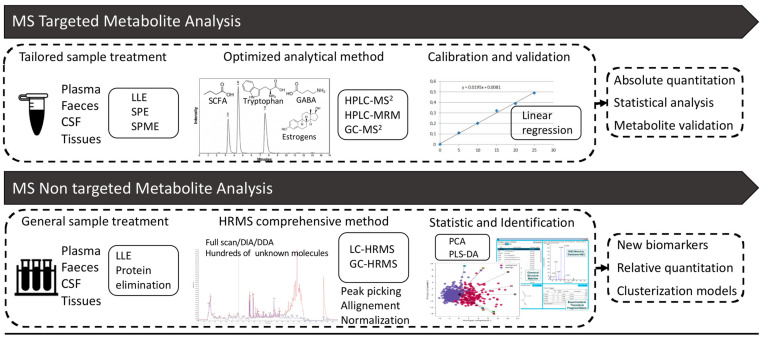
Overview of targeted and untargeted metabolomics workflows using MS. The targeted approach involves specific sample preparation techniques (e.g., liquid–liquid extraction (LLE), solid-phase extraction (SPE), and solid-phase microextraction (SPME)) and optimized analytical methods, typically employing tandem mass spectrometry (MS^2^), for precise metabolite identification and quantification. The untargeted approach utilizes more general treatments (e.g., LLE or protein precipitation) and high-resolution mass spectrometry (HRMS) to detect unknown metabolites. Statistical tools, such as principal component analysis (PCA) and orthogonal partial least squares discriminant analysis (OPLS-DA), support biomarker discovery and clustering pattern identification.

MS-based metabolomics usually proceeds through two main approaches, targeted and untargeted, briefly compared in Figure 2. The targeted approach starts with an a priori hypothesis (hypothesis-driven), optimizing the method for a single molecule or a class of physico-chemically similar molecules. In contrast, the untargeted approach is non-selective and aims to detect the widest possible range of molecules with different characteristics and functions in a new biomarker discovery perspective. It is important to emphasize that these two approaches are not mutually exclusive. On the contrary, they can be used complementarily to provide a more comprehensive understanding of the metabolic landscape [89,90].

For a better understanding of the application of MS coupled with separative techniques for the characterization of stress mediators involved in the MGB axis, we will describe in the next sub-sessions the analytical approaches and the most recent methods grouped for the different biological matrices.

### 3.1. Targeted Mass Spectrometry Analysis

Most methods developed up to about 15 years ago were based on targeted screening (TS) or targeted analysis (TA) techniques. TA studies focus on a small, specific subset of metabolites, typically fewer than 30, although broader targeted studies involving 100 or more metabolites are becoming more common. Analytes are often selected on the basis of shared functions, involvement in the same metabolic pathways, or other biological interests where prior knowledge exists [91].

The approach used in TA can provide quantitative data, such as absolute concentrations for one or more compounds, thanks to the linear concentration–response correlation and the high degree of specificity, sensitivity, precision, and accuracy [92]. This capability is extremely useful in hypothesis testing, where similarities or differences among groups are typically evaluated.

Given the limitations of MS mass measurement alone, additional information is essential for complete characterization. Therefore, a separation technique (e.g., GC, LC, capillary electrophoresis) coupled to a mass analyzer is typically used. In many cases, at least one separation technique is employed, but the most advanced studies utilize multiple complementary separation techniques. This approach, known as a multiplatform analysis, allows for a broader and more detailed exploration of the sample’s complexity [93,94].

In recent years, tandem configurations have become increasingly common. These configurations, which incorporate multiple mass analyzers in sequence, allow the identification of precursor ions by analyzing fragmentation patterns, which are critical for structural elucidation. In addition, tandem MS can effectively overcome common challenges, such as ion suppression and matrix effects [95].

Instruments with a single analyzer are constrained to either full-scan analysis or Selected Ion Monitoring (SIM), focusing on a specific set of ions that are directly generated from the sample. Conversely, instruments with tandem MS capabilities can utilize a variety of acquisition strategies. Among these, Multiple Reaction Monitoring (MRM) is the most prevalent. MRM shifts the focus from monitoring ions resulting from the analyte ionization to monitoring the more diagnostic, intense, or characteristic fragment ions that arise from the analyte after fragmentation [96].

TA methods must be thoroughly developed and optimized and the sample preparation stage in development is often the longest and most technically challenging. The primary objective is to eliminate as many interferences as possible while optimizing the recovery of the analyte(s). In cases where low concentrations are being measured, it may also be advantageous to implement a concentration step. Key techniques, such as vacuum drying, protein precipitation, Solid-Phase Extraction (SPE), and Solid-Phase Microextraction (SPME), play a crucial role in both cleanup and pre-treatment (Figure 2).

Validation of quantitative analysis allows the determination of absolute concentration as well as the validation of new biomarkers [67].

### 3.2. Non-Targeted Mass Spectrometry Analysis

Ongoing technological advances in both chromatography and the production of increasingly high-resolution mass spectrometers (HRMS) have led to the introduction of less selective strategies known as untargeted or Non-Targeted Analysis (NTA) [97,98,99]. NTA allows the monitoring of known, suspected, or still unknown molecules, up to the identification of previously undetected compounds. Despite the specific advantages of these analyses, such as simpler sample and method optimization and the lack of need for chemically similar standards, their widespread application is still limited [100]. This is likely due to the complexity of data analysis and the lack of standardization between the experimental and data analysis protocols of different laboratories.

Untargeted studies are typically employed when the aim is to formulate hypotheses, or when only a broad hypothesis is in place, typically centered around the existence of metabolic variations between cases and controls but without specific knowledge of the nature of these variations [101].

The design of these experiments typically revolves around the detection of as many metabolites as possible, with the ideal goal of covering the entire metabolic network; however, researchers may focus on specific subclasses of metabolites that share common physico-chemical properties, such as in lipidomics or volatilomics [102,103].

NTA studies involve the simultaneous measurement of a large number of metabolites within each sample, resulting in large and intricately structured datasets. Extracting meaningful biological insights from this wealth of information requires a series of essential processing steps that spectra can undergo, either independently or together, culminating in the generation of a definitive set of features [104,105]. In metabolomics analyses, a feature refers to a measurable entity identified in the raw data produced by analytical instruments, characterized by distinctive attributes, including mass-to-charge ratio, retention time, and signal intensity.

After general sample preparation, e.g., solid–liquid (SLE) or liquid–liquid (LLE) extractions, protein precipitation, and LC/GC-HRMS analysis, in a standard methodological pipeline (Figure 2), the first step is to refine the spectral data through baseline correction and noise filtering to remove undesirable peaks present in blank samples or originating from solvent constituents [106]. This is followed by feature detection, commonly referred to as peak picking, using various available tools. These tools, which include open-source options (e.g., Mzmine [107,108], MS-DIAL [109]) and commercial software (e.g., Compound Discoverer, Progenesis QI), employ distinct algorithms tailored to the specific analytical platform utilized.

The need for spectral alignment depends on the analytical setup employed. In multi-sample NTA studies, spectral alignment is typically a critical processing step. Spectral alignment algorithms can be broadly categorized into two main groups: spectral alignment methods, in which spectral data are aligned before peak detection, and peak-based alignment methods, which focus on aligning spectral peaks across samples post-detection using their coordinates in terms of *m*/*z* and retention time [110,111].

A crucial step in ensuring accurate feature analysis in NTA studies is data normalization. The primary goal of normalization is to remove unwanted systematic bias, ensuring the data reflects only biologically meaningful differences. This step is particularly vital when dealing with complex biofluids like blood or feces. Several normalization techniques have been developed to address these challenges, including median normalization, MS total useful signal (MSTUS), median absolute deviation (MAD), and probabilistic quotient normalization (PQN) [112,113,114,115].

In untargeted metabolomics, three main MS data acquisition modes, full-scan, data-dependent acquisition (DDA), and data-independent acquisition (DIA), are employed to record metabolite signals, as shown in Figure 2 [116].

The choice of acquisition techniques depends on the instrumental setup and is particularly influenced by the type of ionization employed, typically either hard ionization (e.g., electron impact) or soft ionization (e.g., electrospray ionization, chemical ionization). Soft ionization techniques, with LC-ESI being the most commonly employed combination, were among the first approaches used in MS-based metabolomics and remain the most widely applied today [117].

The process of metabolite identification (also referred to as annotation) requires information from at least two concordant and independent (or orthogonal) methods. For this reason, early untargeted methods on instruments utilizing soft ionization relied on combining retention time information with the accurate mass (<5 ppm accuracy) of the molecular ion, which allowed the calculation of the compound’s molecular formula [118].

For analyses involving isomeric or isobaric molecules, however, the accurate mass of the molecular ion alone is insufficient to distinguish between compounds. In targeted analyses, where the analytes under investigation are known beforehand, chromatographic separation is often sufficient to resolve such ambiguities. However, in untargeted methods, where the presence of all potential isomers cannot be anticipated, resolving ambiguities requires additional information, which is typically obtained through MS^2^ experiments. Since it is not possible to predict a priori which ions will require further fragmentation for identification, nor to isolate and fragment all ions, an improved and automated data acquisition mode, DDA, was developed.

In DDA, the MS instrument performs full-scan MS and, when certain criteria are met, conducts a specified number of MS/MS acquisitions on the most intense ions before switching back to full-scan MS^1^. This explains the origin of the name: MS^2^ acquisition is dependent on (or triggered by) previously recorded data. Although DDA can theoretically be performed on any tandem mass spectrometer, in practice, Q-TOFs and Orbitraps are predominantly used because only these technologies offer sufficient resolution and acquisition rates for metabolomics analyses [119]. To overcome instrumental limitations, MS and MS^2^ acquisition can also be separated into two discrete analyses. Typically, a full-scan analysis is performed on all samples and, once a list of relevant ions is compiled (either manually or using software), fragmentation is carried out on selected ions in specific representative samples, often QC samples [120,121].

However, a limitation of DDA is that low-abundance metabolic features may not be selected for fragmentation due to intensity-dependent precursor ion selection, which can compromise their identification. To address this issue, an alternative analytical approach called DIA was developed. In DIA mode, all precursors within a selected *m*/*z* range are scanned and consistently fragmented, regardless of their abundance. Unlike DDA, DIA fragmentation is not triggered by precursor ion intensity but is predefined by the user, hence the name “data-independent acquisition”. Depending on how the *m*/*z* ranges are defined and the width of the *m*/*z* fragmentation windows, the technique can have different names. In All Ion Fragmentation (AIF or MS^E^), the entire mass range is fragmented simultaneously. A more selective DIA approach is known as Sequential Window Acquisition of All Theoretical Mass Spectra (SWATH) [122], where the mass range of interest is divided into several subranges. Typically, SWATH scans are performed with Q1 window sizes ranging from 30 to 50 Daltons [123].

Since DIA methodologies do not involve precursor selection (apart from the selected mass range), they theoretically generate MS^2^ spectra for all precursor ions present in the sample, allowing for the identification of analytes at trace-level concentrations and providing the possibility of retrospectively investigating data looking for new metabolites without having to reacquire samples; for comparison, in DDA, if the ion is not among the most intense, it might not be fragmented or detected at all [124,125]. While DIA is an attractive strategy for metabolomics profiling, the complexity of the resulting MS^2^ spectra poses a significant informatics challenge for metabolite identification [126]. The dissociation between precursors and their fragment ions requires sophisticated data deconvolution methods, which are not yet widely available [127].

After generating a comprehensive set of metabolic features with either DDA or DIA, the last two steps in this pipeline are feature annotation or identification and statistical data analysis. Feature annotation involves assigning a tentative biological identity to each signal based on multiple orthogonal sources of information. In the best studies, in addition to identification, a confidence level is also assigned to the annotated features. The spectral database matching with libraries containing the exact mass of precursor ion (MS^1^) and tandem mass spectrum (MS^2^) is typically the first and most used method. However, due to the frequent insufficiency of experimental MS^2^ databases, the use of in silico methods to predict MS^2^ spectra and molecular structures has gained popularity [128].

For data analysis, both univariate and multivariate statistical methods can be applied to achieve two primary objectives: exploring the overall structure of the metabolomics data within the dataset using unsupervised methods, such as principal component analysis (PCA), and identifying relationships between metabolic features and phenotypic data using supervised methods, such as partial least squares-discriminant analysis (PLS-DA) or orthogonal PLS-DA (OPLS-DA) [129,130,131].

These final steps of statistical data analysis and feature annotation have an interchangeable order depending on the priorities of the study. If the primary goal is to prioritize speed, performing statistical analysis before annotation allows focusing on significant metabolites while disregarding less relevant ones. On the other hand, if the focus is on maximizing the quantity of information, annotating all detected features first enables a more comprehensive dataset and facilitates the integration of other biological and clinical data.

### 3.3. Biological Matrices and Sample Pre-Treatment

The small molecule metabolites involved in the MGB axis can be of various natures, including lipids, nucleic acids, organic acids, amino acids, carbohydrates, vitamins, minerals, toxins, and pollutants. As discussed above, some of these can be produced or influenced by GM, which can vary over time in relation to endogenous and exogenous factors. In order to study these metabolites and to gain a better understanding of the functioning of the MGB axis, several challenges need to be overcome, including sample collection and the handling and identification of heterogeneous metabolites [21,55].

Feces, blood, urine, and other biological samples, such as intestinal, brain, and liver samples, are the biological matrices most commonly collected and analyzed for metabolite identification [21].

Basically, the type of pre-treatment varies depending on several factors: (i) the nature and complexity of the sample; trivially, feces are a more difficult matrix to extract than plasma or urine because they are in a semi-solid state and because they do not have a markedly polar character like “aqueous” samples. (ii) The type of analytical approach used; in the TA, the extraction of a metabolite or a class of them is optimized according to their chemical-physical properties in order to maximize the yield for the analytes under examination and, on the contrary, to eliminate the interferents. In the untargeted mode, which requires the detection of the greatest possible number of metabolites, a less selective extraction is preferred. (iii) The analytical method, depending on the type of instruments used; for example, LC-MS or GC-MS, the metabolites after extraction must be injected into the system in a compatible solvent—generally, a volatile solvent for GC-MS and aqueous solvent or water-organic solvent mixtures for LC-MS.

A summary of MS-based metabolomics studies on MGB axis mediators is shown in Table 1.

**Table 1:** MS-based metabolomics studies on mediators of the microbiota–gut–brain axis. For each study, the metabolites, the underlying condition, whether the study was conducted on humans/animals, the type of biological sample, and the analytical method used are reported.

Metabolites	Condition	Origin	Biological Matrix	Analytical Method	References
Neurotransmitters: 5-HT, DA, GABA, ACh, NE, etc.	Bipolar disorder	Human	Serum	LC-MS	Li, Z. et al., 2022 [87]
	Neurodegeneration	Animal	Feces	LC-MS	Tilocca, B. et al., 2020 [78]
	Stress, Parkinson’s disease	Human	Cells, feces, intestinal tissue	GC-MS, LC-MS, DESI-IMS	Luan, H. et al., 2017 [14]
	Stress	Animal/Human	Feces	LC-MS, GC-MS	Iannone, L.F. et al., 2019 [73]
	Stress	Rats	Plasma	LC-MS	Bassett, S.A. et al., 2019 [85]
	CUMS	Rats	Hippocampus	UHPLC- MSMS	Li, J. et al., 2019 [132]
	Neurological disorders	Rats	Brain, colon tissues	DESI-IMS	Hulme, H. et al., 2022 [133]
SCFAs, BCFAs, and derivatives	Stress	Mice	Feces	GC-FID	Wouw, M.v.d. et al., 2018 [19]
	Neurodegeneration	Animal	Feces	LC-MS	Tilocca, B. et al., 2020 [78]
	Bipolar disorder	Human	Serum	LC-MS	Li, Z. et al., 2022 [87]
	Cognitive decline	Human	Serum	UHPLC-MSMS	Neuffer, J. et al., 2022 [134]
	Autism	Human	Tssues, blood, feces	GC-MS, DESI-IMS, MALDI-IMS	Luan, H. et al., 2017 [14]
	Stress	Animal/Human	Feces	LC-MS, GC-MS	Iannone, L.F. et al., 2019 [73]
	CUMS	Mice	Feces	GC-MS	Chen, M. et al., 2024 [15]
	Stress	Rats	Cecal samples	LC-MS	Bassett, S.A. et al., 2019 [85]
	Neurological disease	Rats	Brain tissue	DESI-IMS, MALDI-MS	Hulme, H. et al., 2020 [133]
Metabolites of the tryptophan pathway (tryptophan, indole, kynurenine, 5-HT, and related metabolites)	Stress	Mice	Intestinal, brain tissues	HPLC-ECD	Lyte, J.M. et al., 2020 [56]
	Bipolar disorder	Human	Serum	LC-MS	Li, Z. et al., 2022 [87]
	CUMS	Rats	Feces	UHPLC-TOF-MS	Lv, W.-J. et al., 2019 [58]
	Depression	Human	Blood, brain tissue	LC-MS	Luan, H. et al., 2017 [14]
	Stress	Animal/Human	Blood, cerebrospinal fluid	LC-MS, GC-MS	Iannone, L.F. et al., 2019 [73]
	Alzheimer’s disease	Rats	Brain tissue	HPLC-UV-MS	Pappolla, M.A. et al., 2019 [135]
	Stress	Rats	Brain, gut tissues, blood	UHPLC-MS	Deng, Y. et al., 2021 [136]
Amino acids and biogenic amines	CUMS	Mice	Feces	LC-MS/MS	Chen, M. et al., 2024 [15]
	Stress	Rats	Cecal samples	LC-MS/MS	Xu, M. et al., 2020 [86]
	CUMS	Rats	Feces	GC-MS	Li, J. et al., 2019 [132]
	Cognitive decline	Human	Serum	UHPLC-MSMS	Neuffer, J. et al., 2022 [134]
Lipids	Depression	Rats	Brain tissue	UHPLC-MSMS	Hu, K. et al., 2022 [137]
Non-targeted analysis	Neurological disorders	Mice	Serum, feces, brain tissue	UHPLC-MSMS	Lai, Y. et al., 2021 [74]
	Stress	Rats	Brain, plasma	LC-MSMS	Bassett, S.A. et al., 2019 [85]
	Stress	Rats	Feces	UHPLC-MSMS	Coley, E. et al., 2021 [138]
	CUMS	Rats	Feces	GC-MS	Wang, R. et al., 2023 [139]
	Sleep disorder	Rats	Brain, liver tissues, plasma	UHPLC-MSMS	Vallianatou, C.A. et al., 2021 [140]

DESI, desorption electrospray ionization; ECD, electrochemical detection; FID, flame ionization detector; GC, gas chromatography; HPLC, high-performance liquid chromatography; IMS, imaging mass spectrometry; LC, liquid chromatography; MALDI, matrix-assisted laser desorption/ionization; MS, mass spectrometry; MSMS, tandem mass spectrometry; UHPLC, ultra-high-performance liquid chromatography; UV, ultraviolet.

#### 3.3.1. Fecal Samples

Feces, as the reference sample for GM studies, constitute a complex matrix that includes microbial communities (bacteria, fungi, and viruses), undigested food particles, cellular debris, and metabolites generated through microbial fermentation. Given its complex composition, fecal analysis serves as an essential tool for investigating the gut microbiome and its impact on health and disease, particularly in the context of gastrointestinal, metabolic, and neurological disorders [16,21,55,73,75,85,141].

Treatment procedures may vary depending on the nature of the metabolite(s) to be identified. Different extraction solvent polarity, sample pre-treatment, and clean-up are optimized by researchers, depending not only on the type of metabolites treated but also on the analysis modality—including targeted and non-targeted—and on the purpose of the study. Protein precipitation with cold organic solvents, such as Acetonitrile (ACN), and several extraction, clean-up, and concentration methods, like LLE, SPE, and SPME, represent the typical strategies for sample treatment, regardless of the investigated compounds’ nature. Below, we report some significant examples regarding fecal sample handling for the analysis of metabolites implicated in the MGB axis.

Some studies have used SPME for the targeted extraction of 3-methylindole and indole in fecal samples from children with autism and pervasive developmental disorders [16]. On the other hand, other fecal samples have been freeze-dried for TA of SCFAs in patients with Parkinson’s disease [21]. One of the most widely used methods for analyzing SCFAs in feces involves acid water extraction (SLE) and exploits the intrinsic volatility of acids in neutral form by SPME followed by GC-MS analysis [142].

There are several scientific works in the literature monitoring changes in the fecal metabolome during the development of depressive-like behaviors in rats exposed to CUMS [85,86,87,132,143]. For example, Wang et al. [139] extracted lipophilic metabolites from fecal samples using chloroform then, after adding MeOH/H_2_O solution and grinding, finally performed ultrasonic extraction. The samples were subsequently derivatized by adding BSTFA derivatization reagent and finally analyzed by GC-MS. For hydrophilic compounds, another sample processing procedure involved the homogenization of fecal samples in H_2_O, then followed by centrifugation. The resulting supernatant was added to ACN for protein precipitation [132,139]. On the other hand, Chen et al. [15] treated fecal samples by adding an aqueous solution containing 0.5% phosphoric acid. The extracted fecal metabolites were analyzed directly using GC-MS.

An analytical method based on ultra-high-performance LC and electrospray ionization tandem MS (UHPLC-ESI-MS/MS) has been developed and validated for the TA of 50 neurotransmitters and tryptophan metabolites in serum, feces, and cerebral cortical brain tissues [74]. Regarding the extraction of metabolites from fecal samples, they were added with MeOH/H_2_O (50:50, *v*/*v*) containing a mixture of internal standards. After homogenization and centrifugation, aliquots of the supernatant were dried in a vacuum concentrator and resuspended in appropriate solvents for instrumental analysis.

In addition, a study was conducted to evaluate whether SCFA and BCFA supplementation could counteract the lasting effects of chronic psychosocial stress by analyzing fecal samples. The samples were homogenized with HCl in H_2_O, which acidified the samples, ensuring protonation of the SCFAs prior to analysis, preventing the appearance of split peaks in the chromatograms. After centrifugation for 3 min, the supernatant was transferred to GC vials and analyzed by GC—flame ionization detection (GC-FID) [19].

#### 3.3.2. Plasma Samples

Blood samples, including plasma and serum, are by far the most popular sample sources in the search for metabolites involved in the MGB axis [21].

Much more often than whole blood, plasma is investigated for its content of metabolites circulating in both the gut–brain and brain–gut directions. Plasma, blood deprived of the cellular part, is less complex than fecal samples; in fact, the preparation protocols for blood samples mainly require the precipitation of proteins that influence the metabolomic analysis by prevalently suppressing the small molecule ionization [18,55]. For example, the review published by Konjevodan et al. [16] summarizes the work of other researchers in which a standard extraction/precipitation protocol of mixing plasma with acidic ACN was used or protein precipitation occurred by adding trichloroacetic acid.

Plasma, given its nature as an aqueous solution, is mainly analyzed by LC, as reported at the end of the paragraph, but, for the more apolar components, GC is preferred and the sample preparation methods are consequently tailored for this analytical technique. Very often the extracted compounds are not naturally volatile so they require pre-injection derivatization. As an example, the study by Li et al. [132] used ACN added to plasma for protein precipitation. The samples were subsequently centrifuged and the supernatant was completely dried in a nitrogen concentrator and resuspended in pyridine methoxy amino acid salt solution for analyte subsequent derivatization with N,O-bis (trimethylsilyl)tri-fluoroacetamide containing 1% trimethylchlorosilane. After incubation, samples were analyzed by GC-MS and the results showed that changes in the fecal metabolome significantly contributed to changes in the plasma metabolome and were associated with depressive-like phenotypes in CUMS rats.

Moreover, Li et al. [132] investigated the content of SCFAs in the serum of mice exposed to CUMS. The concentrations of SCFAs (acetic acid, propionic acid, isobutyric acid, butyric acid, isovaleric acid, valeric acid, and hexanoic acid) in the serum were determined by GC-MS. For metabolite extraction, 15% of phosphoric acid was added to each serum sample. After centrifugation at 4 °C, the supernatant was treated for the injection in the GC-MS system.

Several studies on the role of steroids in the MGB axis reported the quantitation of the steroid hormone corticosterone in plasma samples using an ELISA kit [57,74,86,87]. ELISA stands for “enzyme-linked immunosorbent assay”. These commercial kits, according to the manufacturers’ instructions, require no sample preparation procedures or simple dilution so they are easy to use. On the other hand, they have the disadvantage of being less versatile, selective, and adaptable. Lyte et al. [56] conducted a study in GM-deficient mice to assess whether the serotoninergic response to acute stress exposure was dependent on the GM community. Numerous biological samples were collected, including blood, intestinal samples, and brain regions. Plasma was used to assess the corticosterone response to acute stress; it was diluted in the dilution buffer provided by the ELISA kit and analyzed by reading the absorbance at 450 nm. The ELISA analytical method is out of the scope of this review but it is still worth mentioning the above works because they are important for the study of GM-related stress mediators.

As mentioned above, given the nature of the plasma and serum matrices, many metabolomics analyses involved LC combined with different MS analyzers or, less often, capillary electrophoresis combined with MS (CE-MS) [14].

In a targeted metabolomics study, focusing on metabolites known to be produced or influenced by GM, including aromatic and derived amino acids, biogenic quaternary amines, secondary bile acids, B vitamins, SCFAs, and derived metabolites, serum samples were first subjected to protein precipitation with MeOH containing 0.1% formic acid, followed by extraction with ACN. The analyses were carried out by UHPLC-MS/MS [134].

In another UHPLC-ESI-MS/MS metabolomics study for neurotransmitter analysis, serum metabolites were extracted with MeOH containing the internal standard. Then, the samples were vortexed, incubated at −20 °C for 30 min, and centrifuged to precipitate proteins and particulates. Aliquots of the supernatant were subsequently dried in a vacuum concentrator and resuspended in appropriate solvents for instrumental analysis [74].

Other researchers followed a similar extraction method for MS-based metabolomics in mice subjected to chronic stress to study the levels of neurotransmitters involved in the MGB axis [136].

Another untargeted metabolomic study was conducted in bipolar disorder patients using LC-MS to reveal potential signaling pathways from the GM to the gut and brain, which may play a role in the pathophysiology of bipolar disorder [60]. Serum samples were mixed with 80% MeOH, vortexed, and centrifuged at 4 °C for protein precipitation. The supernatant was diluted and injected into the liquid chromatograph.

Finally, whole blood samples were processed; as described by J. Yao et al., firstly, they obtained serum, which was added to an internal standard solution. After protein precipitation with cold ACN, the mixture was vortexed and centrifuged and the supernatant was dried under vacuum. The residue was re-dissolved in sodium bicarbonate buffer and DNS-Cl for derivatization. After evaporation, the sample was subjected to solid-phase extraction, eluted with MeOH, and concentrated. The final extract was analyzed by UHPLC-MS/MS [144].

#### 3.3.3. Urine Samples

Urine is another valuable source for the identification of biomarkers for the study of the MGB axis as it reflects the metabolomic state of GM [21]. Sample treatment requires correct sample management due to the high concentration of urea and salts and also correct standardization of the metabolic content due to differences in dilution volume.

To date, only a few studies have used this biological matrix to study the MGB axis under stress conditions. Among them, one study reported the extraction of metabolites by adding a cold MeOH/ACN/H_2_O (5:3:2, *v*/*v*/*v*) mixture to urine and centrifuging. Subsequently, the urine volume was adjusted to obtain 1 M creatinine to avoid detection problems due to excessive dilution or saturation. Finally, another study reported evaporation of the urine sample and resuspension in a smaller volume of hexane to preconcentrate the mostly lipophilic analytes and, at the same time, eliminate insoluble salts [16].

Urine samples can be directly analyzed by headspace, or SPME combined with GC-MS as a powerful technique for detecting the volatile metabolome from the characteristic odor of urine, which has been associated with significant development of Alzheimer’s disease problems in animal models [145,146,147].

#### 3.3.4. Other Biological Samples

The cerebrospinal fluid (CSF) metabolome is more physically connected to the brain than any other fluid. Therefore, it is possible to discover biomarkers of neurodegenerative diseases, improving therapeutic research and understanding of disease pathogenesis. However, studies of the CSF metabolome are still lacking. It has been found that CSF metabolites are significantly associated with the progression from mild cognitive impairment to Alzheimer’s disease. In addition, CSF samples were collected from pathologically verified Parkinson’s disease patients and analyzed using UHPLC and GC coupled to various MS platforms. New biomarkers have been identified for the recognition of excitotoxicity and oxidative stress in the pathogenesis of Parkinson’s disease [14]. Compositionally, CSF is not a complex matrix, containing smaller amounts of proteins, cellular matter, and other interferences than plasma or serum.

Hippocampal samples have also been used, e.g., in the study by Jianguo et al. based on UHPLC-ESI-MS/MS, to evaluate the relationships between depression and metabolic disorders [132]. In particular, 12 neurotransmitters were identified using rat hippocampal samples homogenized and precipitated with MeOH.

Another study by Lai et al. used MeOH to extract metabolites from brain tissue. Specifically, samples were homogenized with MeOH and incubated at −20 °C. After centrifugation, the supernatant of the brain extracts was collected and subjected to evaporation. Finally, the extracts were resuspended in appropriate solvents for UHPLC-ESI-MS/MS analysis [74].

A study was also conducted on the changes in lipid metabolite levels in cerebral tissues—including the regions of the prefrontal cortex and hippocampus—and on GM characteristics in ApoE^−/−^ mice under the co-morbid disease state of depression and atherosclerosis. The metabolites were extracted from each starting sample by adding an extraction solution consisting of dichloromethane (DCM)/MeOH. The samples were sonicated and left at −20 °C for 1 h. The supernatant was centrifuged at 4 °C, evaporated, re-dissolved with a mixture of IPA/ACN/H_2_O (2:1:1, *v*/*v*/*v*), vortexed, sonicated in a 4 °C H_2_O bath, and finally centrifuged at 4 °C. The extracted metabolites were detected using an UPLC-HRMS system [137].

Lyte et al. [56] also analyzed intestinal and brain samples using HPLC coupled with an electrochemical detector (ED). Briefly, the extraction method consisted of sample homogenization in buffer, sonication, and centrifugation at 4 °C. The supernatant was then collected and analyzed by HPLC-ED.

Another study by Xu et al. [86] used blind samples to investigate whether GM and its metabolic functions were altered under stress. Metabolites were extracted from cecum samples by adding ice-cold MeOH/CHCl_3_ (2:1, *v*/*v*). After ice homogenization, the samples were centrifuged and the supernatant was collected for phase separation; additionally, 0.5 mL of the polar phase was then concentrated by evaporation of the solvent in a concentrator. A mixture of 50% ACN and 50% H_2_O was used to reconstitute the metabolites in the LC vials and the samples were then analyzed using HPLC-HRMS.

Finally, a study of the hepatic metabolism of rats after fecal microbiota transplantation identified 74 hepatic metabolites that were differentially expressed between the FMT group and the control group [58]. This study showed that the GM contributed to host liver metabolism and brain inflammation, becoming a potential cause of depression. Liver tissue samples were homogenized with ultrapure H_2_O and then a mixture of MeOH/ACN (1:1, *v*/*v*) was added for vortexing and sonication for 30 min. After centrifugation, the supernatant was analyzed by UHPLC combined with MS using a Triple TOF mass spectrometer.

## 4. Multi-Omics Correlations: From Traditional to Cutting-Edge Data Integration Approaches

**Figure 3:**
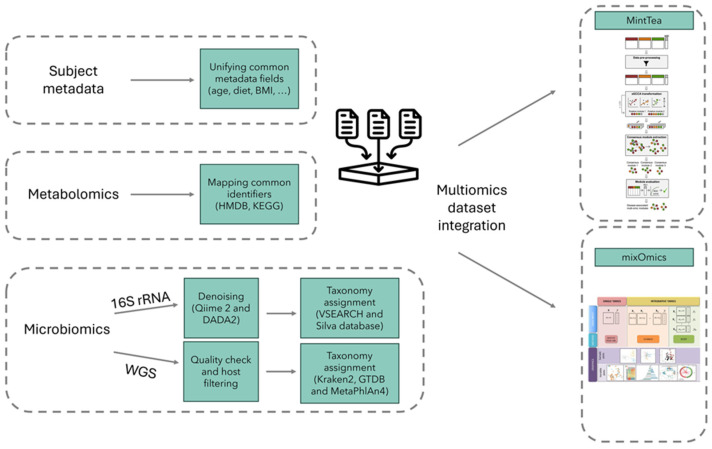
Multi-omics integration. An overview of data resources, key processing steps, and main bioinformatics tools for a microbiome/metabolome dataset is shown. Multi-omics integration can be performed using several tools, such as MintTea [166] and mixOmics [167]. The microbiome can be profiled using 16S rRNA amplicon sequencing or whole-genome sequencing (WGS). BMI, body mass index; HMDB, human metabolome database; KEGG, Kyoto Encyclopedia of Genes and Genomes.

An increasing number of studies are now available that have used multi-omics approaches, such as metagenomics and metabolomics, with the aim to evaluate the relationship between different players (e.g., microbes/genes and metabolites) in different clinical settings, potentially providing mechanistic insights. To identify potential associations and common patterns in large multi-omics datasets, advanced computational methods and appropriate statistical tools are essential (Figure 3). For example, integrated microbial/metabolic signatures can be identified using univariate methods, such as Pearson, Spearman, or Kendall correlation tests, and/or multivariate methods that consider potential dependencies between features, such as PLS, linear regression, and multidimensional scaling (MDS) [148,149,150,151]. Adjustments for age, sex, body mass index, or other relevant host metadata should be considered. More recently, multi-omics integration has been performed using machine learning (ML) approaches by first training a ML model to predict a given phenotype based on all features and then using various model explanatory methods to detect informative features in the ML model [152,153,154,155,156]. This type of analysis is even more relevant when validating findings across studies and cohorts to provide a biologically meaningful fingerprint of a given hypothesis [157,158]. However, obtaining and processing multi-omics datasets from different studies remains extremely challenging and time consuming, particularly due to the difficulty of retrieving complete and standardized data from open-access repositories.

In untargeted metabolomics, even integrating results from different platforms can be particularly challenging. These difficulties arise from the wide variety of instrumental setups used globally. Key factors that can differ significantly include the separation technique (e.g., GC, LC, flow injection), the analytical method (e.g., flow rates, column types, ionization modes), the detector type (e.g., quadrupole, ion trap, TOF), and even the specific software settings used to convert raw data into biochemical insights [159].

Integrating metabolomics data with data from other -omics analyses is even more complex, heavily depending on the available datasets, sample characteristics, and study objectives. Issues such as data dimensionality, heterogeneity, sample depletion, and the lack of universally adopted protocols add further difficulties.

Organizations such as the Metabolomics Quality Assurance and Quality Control Consortium, the International Lipidomics Society, and the International Metabolomics Society are actively advancing interlaboratory studies, best practice guidelines, reference materials, and data standards [160,161,162,163]. These efforts aim to enhance data quality in metabolomics datasets and drive the harmonization of metabolomics workflows.

In this context, data curation and bioinformatics expertise are critical. For example, integrated multi-omics datasets can be processed with a combination of random forest regressor models and random-effects models to find robust associations across studies, which can then be visualized using bipartite networks [158,164,165].

To facilitate these analyses, several tools have been developed and made available. One example is the recently published “MintTea” pipeline for the analysis of multi-omics microbiome data [166]. This tool is based on the hypothesis that each disease-related microbial mechanism may involve different taxa, functions, and metabolites that collectively act in the disease landscape; therefore, MintTea aims to identify robust “disease-associated multi-omic modules”, each of which comprises a set of features from the different omics that exhibit coordinated variation across omics and, as a whole, are associated with the disease or phenotype of interest. Another example of a statistical tool commonly used for multi-omics analysis is “mixOmics”, with a special focus on data exploration, dimension reduction, and visualization through easy-to-read PCA plots, networks, heatmaps, and circos plots [167].

Despite this progress, the road is still uphill, with several wet and in silico challenges. For example, as mentioned above, multi-omics integration requires extensive data curation, bioinformatics, and biostatistics work. Furthermore, the use of 16S rRNA amplicon sequencing versus whole-genome sequencing (WGS), and differences in library preparation and sequencing depth, can greatly affect the resolution and accuracy of the obtained microbiome profiles. In metabolomics, different platforms targeting different chemical classes and non-standard workflows make it difficult to integrate studies; nonetheless, efforts from different consortiums and societies are actively enhancing data quality in metabolomics datasets and the harmonization of workflows [168,169]. No less importantly, multi-omics integration results in a list of features associated with the phenotype of interest, without revealing the underlying mechanisms, which need to be investigated in dedicated studies.

## 5. Conclusions

Metabolites mediating MGB axis communication have been extensively studied in relation to neurodegenerative diseases and severe conditions, such as stroke/brain injury, due to their progressive and disabling nature. Conversely, studies aimed at characterizing mediators of the above-mentioned axis that influence stress states have been less reported. The study of mediators of the MGB axis has mainly been carried out using targeted chemical analyses, including MS-based metabolomics. Many of these mediators, such as neurotransmitters, are indeed known to be active in various metabolic pathways involved in pathologies or atypical conditions of the CNS. In contrast, the non-targeted approach for the discovery of novel molecules able to modulate stress resilience or vulnerability via the MGB axis is still largely unexplored.

Since stress has recently been recognized as a disabling condition and is of social interest, in-depth scientific research at the molecular level should be encouraged. The main obstacles to progress in this field are basically the difficulty and high cost of the analyses and the availability of reproducible and controlled animal models or human cases.

With this review, the authors aimed to provide an overview of the contribution of the MS-based metabolomics approach in shedding light on the complex molecular mechanisms/mediators involved in the MGB axis, particularly impacting mental distress generally defined as stress. In this context, we considered the main mediators and their effects, the biological matrices in which they can be determined, and the analytical approaches tailored for this study.

## Data Availability

No new data were created or analyzed in this study.
